# A Professorial Thersites

**Published:** 1850-05

**Authors:** 


### THE WESTERN JOURNAL.

THIRD SERIES.—VOL. V.—NO. V.

LOUISVILLE, MAY 1, 1850.

### A PROFESSORIAL THERSITES.

A character of this kind has recently taken us to task for not conducting this Journal in a way to please him, and, as a specimen of his "good breeding" and veracity, undertakes to make his readers believe that we had written the word "unbeknown" in our article on the bandage, when he knew it was quoted from the writings of a gentleman who published the report of a case, in 1838.

The quarrelsome genius, to whom we refer, is in great agony because his Agamemnon has received some fancied slight at our hands. We owe the spirit of the scolding neither excuse, explanation, nor apology. But our exemplar seems to feel that he owes a disclaimer of any connection between his idol, and a malignant, vulgar, billingsgate specimen of slang that recently adorned a medical journal, which is now in the hands of our termagant. The disclaimer, however, amounts to nothing—*we know that it is false*.

But it is strange that after showering his malice and vituperation upon us, for not kneeling as low at the shrine of his idolatry, as he does, and after cackling at a terrible rate over a word, quoted from an article printed twelve years ago, our scold permits his idol to make a singular display of his classics, in a medical journal, under the control of "the defender of the faith." If the reputation of the idol were half as dear to our doughty champion, as he pretends it is, he certainly would have paid some attention to the article on fractures, in the April No. of his Journal, and would not have permitted the article to appear in a style, in which typographical blunders, grammatical errors, and rhetorical sins strive for the mastery. We should not have attempted the ungracious task of pointing out these things, if the work had not been forced upon us as an act of self defence; and, even now, we shall confine our references to those blunders that lie upon the surface.

As a specimen of the typographical errors, the word bony is repeatedly spelt "boney." The entire essay is studded with typographical blunders, which an editor should have corrected, in an article written by one for whom he pretends to feel a prodigal esteem.

But grammatical errors also abound. On page 430, "removal and reapplication" are made antecedents to "this." The first sentence of a distinct paragraph, on page 438, reads thus: "*Fifteen days* transpired before *they* became so loose as to render it necessary to remove and reapply them, after the same manner." How did the fifteen days become loose, and how were they removed and reapplied? If "they" does not refer to the fifteen days, to what does it apply? And it must be a matter of curious inquiry how either one day or fifteen days could, by any possibility "transpire." Days have no epidermis for excretions to pass through. Transpire is strictly a medical term, and, as such, always means an excretion from the skin. When not used in its medical sense, transpire means the passage of any thing from a state of secresy to publicity, and it never can be used with any, the least approach to correctness, except in one of these two meanings. It would be just as proper to say that fifteen days sailed or rode on horseback, as to say they transpired. If our correspondent, "C.," so invidiously referred to by our assailant, has any special fondness for these things, he must be something more than a glutton, if he desires a more liberal feast than he can find in this recent repast. He may gather his pleasures here, as he would mushrooms in an autumnal pasture. On page 441, we read, "*one set* of muscles only *are* placed," &c. Again: "*its* mischievous effects," &c., "*is* familiarly known." On page 444, we have it: "as the disorganized *parts is* reduced." On page 450, we are told: "*a light regimen*, with a free state of the bowels, *were* enjoined." On page 432, we have an account of a patient, who, while on his way from

Lexington to a neighboring State, re-fractured his patella, and we are told: "Returning to town, after encountering some delay, I found the limb," &c. This would seem to show that the surgeon went to the country, and returned to town; when the writer was trying to say, that the patient returned to town.

Throughout the article, we find, scattered with a prodigal hand, such phrases as these: "Conclusion of the period," "conclusion of the second week," "conclusion of the sixth week," "conclusion of the fourth week." We should think that the merest tyro in philology would know that conclusion, in matters of time, cannot be used as a synonyme of end. The literal meaning of conclusion is the winding up of all arguments and reasoning, not the winding up of a week, or of any period of time, nor can it be correctly used in any other than its etymological sense.

This is but a portion of the classicalities referred to, and if a man's friends will thus treat him, what must he expect from his enemies? If any man ever had just reason for wishing to be saved from his friends, it is the victim of the bungling friendship before us. If the conductor of the Journal to which we allude, were as chary of the reputation of his idol as he pretends to be, he would not have permitted him to appear before the public in such a gala suit as we have described.

Another colleague of our scold appears in the same Journal, of which that colleague is also a conductor, in an article steeped in writing that would disgrace a schoolboy. We shall not dissect this rhetoric, but shall be content with a solitary specimen. On page 455, this lucid and elegant sentence occurs: "We are informed that important and fearful hemorrhages do not always immediately succeed to injuries of the kind, *but that they yet more times occur afterwards*," &c.

We think one specimen of that style is sufficient.

